# End of the Ebola virus outbreak: time to reinforce the African health system and improve preparedness capacity

**DOI:** 10.11604/pamj.2016.23.121.8955

**Published:** 2016-03-24

**Authors:** Ivy Mulinge, Kenneth Soyemi

**Affiliations:** 1 Department of Pediatrics, John H Stroger Hospital of Cook County, 1901 W Harrison Street, Chicago, IL 60612, United States

**Keywords:** Ebola outbreak, health systems preparedness, return on investment

## Brief

The West African Ebola (EBV) outbreak that spanned two years infected 30,000 people with case mortality rate of 36%. Of the 11,000 deaths, 500 (4.5%) were Health Care Workers (HCWs). Majority of HCWs deaths occurred in the initial months of the outbreak depleting the active HCWs workforce in affected countries [1, 2]. Rapidity of the epidemic in the affected countries outpaced the response, and as a result infection and mortality rates rose exponentially in the initial phase of the outbreak. Health systems and local economies were stretched, and it is estimated by the World Bank, that forgone income across the three countries in 2014-2015 were over $2 billion [3].

This outbreak had significant public health impact because for the first time (despite EBV recurring in African countries since the early seventies), the outbreak occurred in an urban setting leading to the rapid dissemination. Factors documented from previous studies that contributed to the epidemic include environmental features, poor human resources, infrastructure deficits, and residence in areas of high poverty [4]. Multinational and multi-organizational response assistance came in the form of technical expertise (Infection Control, diagnostics laboratory aid, epidemiological and clinical support) and financial assistance to the tune of billions of dollars. Response to the outbreak although late was effective in containing the virus which lead the World Health Organization (WHO) on 29 December 2015, to declare the end of the EBV outbreak [5].

In Africa, the health infrastructure is fragile (except in a few countries) and generally is not capable of handling an unexpected or sudden increase in surge capacity such as experienced in the EBV epidemic. The combination of high African HCWs EBV related mortality and previous low estimates of HCW density (2.7 physicians per 10,000 population (range of 0.01-0.2 in the affected countries) and 12.4 nurses and midwives per 10,000 population (range of 1.7-2.7 in the affected countries)) [6] validates the need for HCWs training to replenish the depleted workforce. The diminished density combined with intended medical student exportation, (as more than 40% of African medical students in a previous survey intend to do residency training abroad after graduating medical school) are recipes for worsening of the medical brain drain [7]. In 2001, African Union countries pledged to commit 15% of their national budgets to health spending. Few countries have fulfilled that pledge thus making most health systems weak and still vulnerable to a novel epidemic [8]. African governments should use the current low per capital health spending, rapid globalization, and the likelihood of a similar outbreak in the future, as the impetus to improve preparedness capability by making positive fiscal budgetary changes, and the gross domestic product (GDP) reallocation.

The recently released report of the Harvard-LSHTM Independent Panel on the Global Response to Ebola by Suerie Moon et al. illuminates several essential reforms necessary to prevent future epidemics and consequently pandemics These reforms target the following three areas: 1) Prevention, 2) Response of major disease outbreaks, and 3) Research which encompasses production and sharing of data, knowledge and technology [9]. Ability to optimize response to an incident requires series of coordinated and synchronized responses that intertwines core components of disease response such as tele-communications, Information Technology, road and transportation network, epidemiology and laboratory capacity, crisis communication, Infection control practices and human capital development. To address ongoing infrastructure problems governments should use the EBV after-action Reports to create customized SWOT and GAP analysis; increase per-capital spending and use the results of the aforementioned analysis to bolster financial funding for public health services and health systems development. The low hospital infrastructural density should ignite hospitals development across the region; planning should be supported by con-current analysis of population density data, transportation network analysis, and improved tele-communications infrastructure. African governments should as part of preparedness capacity building invest in equipping hospitals with Personal Protective Equipment (PPE) and basic health care resources which include protective gloves and gowns, intravenous fluids, adequate sanitation, and clear health protocols and guidelines as noted in a previous study to have been deficient in most areas where the EBV outbreak occurred [10].

As health care professionals from developing countries, we ask our governments to invest in the core components of disaster preparedness and capacity building so that countries are self-sufficient and prepared to respond promptly in future responses instead of waiting for external help. The return of investment (ROI) in public health infrastructure strengthening include accelerated economic development, prompt response capability, improvement in life expectancy, increase economic activity and growth, saving of lives of millions of Africans and prevention of disabilities. It is documented that healthy individuals are more productive, earn more, save more, invest more, consume more, and work longer, leading to a positive impact on GDP of a nation. These ROI factors should motivate African governments to start planning and re-enforcing health systems now.

## References

[1] World Health Organization Health worker Ebola infections in Guinea, Liberia and Sierra Leone A PRELIMINARY REPORT.

[2] Stephen Morrison J (2015). After the Ebola catastrophe.

[3] World Bank Update on the Economic Impact of the 2014 Ebola Epidemic on Liberia, Sierra Leone, and Guinea.

[4] Alexander KA SC, Marathe M, Lewis BL, Rivers CM, Shaman J (2015). What Factors Might Have Led to the Emergence of Ebola in West Africa?. PLoS Negl Trop Dis..

[5] World Health Organization (2015). Declaration of Dr. Mohammed Belhocine, head of WHO country office, to mark the end of Ebola Outbreak in Guinea.

[6] World Health Organization (2015). World health statistics 2015.

[7] Bailey N, Mandeville KL, Rhodes T, Mipando M, Muula AS (2012). Postgraduate career in-tentions of medical students and recent graduates in Malawi: a qualitative interview study. BMC medical education..

[8] KPMG International (2012). THE STATE OF HEALTHCARE IN AFRICA.

[9] Moon S, Sridhar D, Pate MA, Jha AK, Clinton C, Delaunay S (2015). Will Ebola change the game? Ten essential reforms before the next pandemic: the report of the Harvard-LSHTM Independent Panel on the Global Response to Ebola. Lancet..

[10] Boozary AS, Farmer PE, Jha AK (2014). The Ebola outbreak, fragile health systems, and quality as a cure. JAMA..

